# Prevalence of transthyretin cardiac amyloidosis in patients with high-degree AV block

**DOI:** 10.1136/openhrt-2024-002606

**Published:** 2024-03-27

**Authors:** Douglas Cannie, Kush Patel, Alexandros Protonotarios, Imogen Heenan, Athanasios Bakalakos, Petros Syrris, Leon Menezes, Perry M Elliott

**Affiliations:** 1 University College London, London, UK; 2 St Bartholomew’s Hospital, London, UK

**Keywords:** Bradycardia, Cardiomyopathy, Restrictive, Pacemaker, Artificial, Epidemiology

## Abstract

**Objective:**

Transthyretin amyloid cardiomyopathy (ATTR-CM) is an infiltrative cardiac disorder caused by deposition of wild type or mutated transthyretin. As ATTR-CM is associated with conduction disease, we sought to determine its prevalence in patients with idiopathic high-degree atrioventricular (AV) block requiring permanent pacemaker (PPM) implantation.

**Methods:**

Consecutive patients aged 70–85 years undergoing PPM implantation for idiopathic high-degree AV block between November 2019 and November 2021 were offered a 3,3-diphosphono-1,2-propanodicarboxylic acid (DPD) scan. Demographics, comorbidities, electrocardiographic and imaging data from the time of device implantation were retrospectively collected.

**Results:**

39 patients (79.5% male, mean (SD) age at device implantation 76.2 (2.9) years) had a DPD scan. 3/39 (7.7%, all male) had a result consistent with ATTR-CM (Perugini grade 2 or 3). Mean (SD) maximum wall thickness of those with a positive DPD scan was 19.0 mm (3.6 mm) vs 11.4 mm (2.7 mm) in those with a negative scan (p=0.06). All patients diagnosed with ATTR-CM had spinal canal stenosis and two had carpal tunnel syndrome.

**Conclusions:**

ATTR-CM should be considered in older patients requiring permanent pacing for high-degree AV block, particularly in the presence of left ventricular hypertrophy, carpal tunnel syndrome or spinal canal stenosis.

**WHAT IS ALREADY KNOWN ON THIS TOPIC**

- There is a high prevalence of transthyretin amyloid cardiomyopathy (ATTR-CM) in patients with common presentations of cardiac disease.

**WHAT THIS STUDY ADDS**

- There is an 8% prevalence of ATTR-CM in older patients requiring a pacemaker for high-degree atrioventricular (AV) block. Patients with ATTR-CM exhibited known red flag features of the disease such as left ventricular hypertrophy, carpal tunnel syndrome or spinal canal stenosis.

**HOW THIS STUDY MIGHT AFFECT RESEARCH, PRACTICE OR POLICY**

- A high-degree of clinical suspicion should be maintained for a diagnosis of ATTR-CM in older patients requiring a pacemaker for high-degree AV block, particularly in the presence of known red flag features of the disease.

## Introduction

Transthyretin amyloid cardiomyopathy (ATTR-CM) is an infiltrative disease of the myocardium caused by deposition of wild-type or mutated transthyretin amyloid fibrils in the extracellular space. Left untreated, the median survival for patients with ATTR-CM_wt_ is 3.6 years^1^ and 2.6 years for those with ATTR-CM_m_ caused by the most common genetic variant, p.Val142Ile.^2^ Treatment with tafamidis, a novel non-NSAID (non-steroidal anti-inflammatory drug) benzoxazole derivative that selectively binds the thyroxine binding sites of the transthyretin tetramer inhibiting its dissociation,^3–7^ has been shown to reduce the risk of death and cardiovascular-related hospitalisations and to slow the decline in functional capacity and quality of life.^8^ Therefore, early diagnosis of ATTR-CM is essential to identify patients who would benefit from disease-modifying therapy.

ATTR-CM is characterised by increased left ventricular (LV) wall thickness, impairment of left ventricular (LV) systolic and diastolic function and conduction abnormalities necessitating permanent pacemaker (PPM) implantation.^9^ Recent studies have shown a high prevalence of ATTR-CM in patients with common presentations of cardiac disease. For example, ATTR-CM was found in 15% of patients with heart failure with preserved ejection fraction and 13% of individuals with aortic stenosis undergoing transcatheter aortic valve implantation (TAVI).^10–13^ The aim of this study was to determine the prevalence of ATTR-CM in older patients undergoing PPM implantation for high-grade atrioventricular (AV) block.^14^

## Methods

This was a single-centre, prospective study with participating patients providing written informed consent.^15^ Patients or the public were not involved in the design, conduct or reporting plans of the study.

Device implantation records at Barts Heart Centre were used to identify patients aged 70–85 years who had required PPM implantation for high-degree AV block between November 2019 and November 2021. Patients were excluded when high-degree AV block was attributable to myocardial infarction, recent cardiothoracic surgery or TAVI. Patients were also excluded when comorbidities precluded travel for nuclear scintigraphy or where patients did not have capacity to consent.

Consenting patients underwent nuclear scintigraphy with 99mTechnetium (Tc)- labelled 3,3-diphosphono-1,2-propanodicarboxylic acid (DPD) at Barts Heart Centre with results reported by local specialist radiologists. Demographics, comorbidities, rhythm and imaging data were retrospectively collected. A positive DPD scan was defined as a Perugini grade 2 or 3.^16^ Where a positive result was returned, patients were referred into a specialist cardiomyopathy service for further investigation including exclusion of light chain (AL) amyloidosis (with serum and urine electrophoresis and serum free AL assays) and *TTR* gene sequencing.

All data were anonymised and statistical analyses performed using the Python programming language (V.3.8, Python Software Foundation).^17^ Continuous variables were tested for normality of distribution by visual inspection of histograms and statistical normality tests (Shapiro-Wilk). Normally distributed variables are expressed as mean±SD and non-normally distributed variables as median (25th, 75th percentiles). Categorical variables are reported as counts and percentages, as appropriate. The *TableOne* library was used for the construction of summary statistics tables and for all statistical comparisons.^18^

## Results

355 patients aged 70–85 years requiring PPM implantation for high-degree AV block within the recruitment period were identified. Following case note review, 162 patients were approached to participate in the study. 39 patients (79.5% male with mean (SD) age at device implantation of 76.2 (2.9) years) had a DPD scan. Remaining patients declined to participate, with most citing frailty in combination with anxiety concerning COVID-19. Three of 39 patients undergoing a DPD scan (7.7%, all male) had a result consistent with a diagnosis of ATTR-CM. All remaining scans were Perugini grade 0. Table 1 shows baseline characteristics stratified by the result of DPD scan.

**Table 1:** Baseline features of patients undergoing permanent pacemaker implantation for high degree atrioventricular block, stratified by outcome of nuclear scintigraphy

	Overall	Negative DPD	Positive DPD	P value
n	39	36	3	
Sex, n (%)	31 (79.5)	28 (77.8)	3 (100.0)	1
Age at PPM implant, mean (SD)	76.2 (2.9)	75.9 (2.6)	80.5 (3.7)	0.2
White ethnicity, n (%)	33 (84.6)	30 (83.3)	3 (100.0)	1
LV internal diameter (mm), mean (SD)	49.6 (7.6)	49.9 (7.4)	46.7 (10.4)	0.6
Left atrial diameter (mm), mean (SD)	40.0 (6.1)	39.8 (6.2)	41.3 (5.9)	0.7
LV ejection fraction (%), median(Q1, Q3)	57.5 (52, 61.0)	57.5 (54, 60.5)	39.0 (31, 51)	0.2
Maximum LV wall thickness (mm), mean (SD)	12.1 (3.4)	11.4 (2.6)	19.0 (3.6)	0.06
Coronary artery disease, n (%)	3 (7.7)	2 (5.6)	1 (33.3)	0.2
Severe valve disease, n (%)	1 (2.6)	1 (2.8)	0	1
Diabetes mellitus, n (%)	10 (25.6)	9 (25.0)	1 (33.3)	1
Hypercholesterolaemia, n (%)	25 (64.1)	23 (63.9)	2 (66.7)	1
Hypertension, n (%)	21 (53.8)	20 (55.6)	1 (33.3)	0.6
Ever smoked cigarettes, n (%)	23 (59.0)	22 (61.1)	1 (33.3)	0.6
Stroke, n (%)	1 (2.6)	1 (2.8)	0	1
Heart failure, n (%)	4 (10.3)	3 (8.3)	1 (33.3)	0.3
Previous atrial fibrillation, n (%)	10 (25.6)	10 (27.8)	0	0.6
Previous NSVT, n (%)	1 (2.6)	0	1 (33.3)	0.08
Third degree AV block, n (%)	24 (61.5)	22 (61.1)	2 (66.7)	1
Carpal tunnel syndrome, n (%)	4 (10.3)	2 (5.6)	2 (66.7)	0.02
Spinal canal stenosis, n (%)	3 (7.7)	0	3 (100.0)	<0.001

Negative DPD scans were those with Perugini grade 0 and positive scans were Perugini grade 2 or 3.

All patients who did not have 3rd degree AV block had fixed 2:1 AV block.

AV, atrioventricular; DPD, 3,3-diphosphono-1,2-propanodicarboxylic acid; LV, left ventricular; NSVT, non-sustained ventricular tachycardia; PPM, permanent pacemaker.

**Figure 1:**
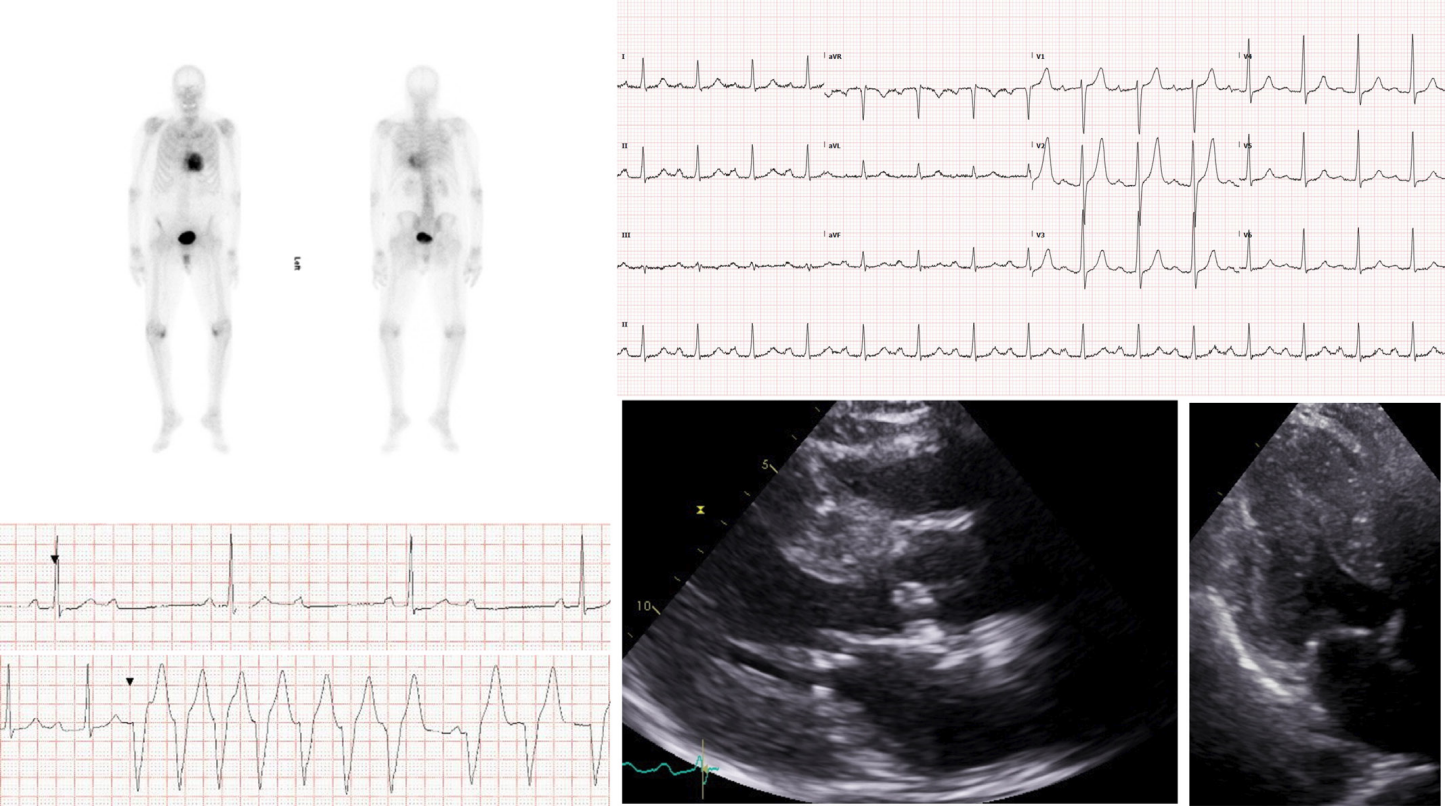
Investigations for a male in his ninth decade requiring ICD implantation for 2:1 AV block and ventricular arrhythmia. Perugini grade 3 uptake on DPD scan (top left) consistent with a diagnosis of transthyretin amyloidosis cardiomyopathy. ECG showing low voltage QRS complexes in limb leads and first-degree heart block (top right). Traces from Holter monitoring showing 2:1 fixed AV block and non-sustained ventricular tachycardia (bottom left). Echocardiography showing concentric left ventricular hypertrophy and near complete cavity obliteration in systole (bottom right). AV, atrioventricular; DPD, 3,3-diphosphono-1,2-propanodicarboxylic acid; ICD, implantable cardioverter defibrillator.

Mean (SD) maximum LV wall thickness of patients with a positive DPD scan was 19 mm (3.6 mm) vs 11.4 mm (2.7 mm) in those with a negative scan (p=0.06). All three patients who returned a positive DPD result had a history of spinal canal stenosis and two had carpal tunnel syndrome. All three had a wild-type genotype. Clinical features of patients with a positive DPD scan are shown in table 2 and figure 1.

**Table 2:** Features of patients with Perugini grade 2 or 3 on DPD scan

	Patient 1	Patient 2	Patient 3
Sex, n (%)	Male	Male	Male
Comorbidities	Hypothyroidism	MI, T2DM, prostate cancer	Hypertension
Age at device implantation (years)	84.8	78.6	78.1
LV internal diameter (mm)	35	55	50
Left atrial diameter (mm)	37	48	39
LV ejection fraction (%)	62.5	22.5	39
Maximum LV wall thickness (mm)	20	15	22
Previous arrhythmia	NSVT	Nil	Nil
NTproBNP (ng/L)	983	3462	626
Perugini grade	2	2	3
Degree of heart block	Fixed 2:1	Third degree	Third degree
Light chain amyloidosis screen	Negative	Negative	Negative
Genotype	Wild-type	Wild-type	Wild-type
Carpal tunnel syndrome	Yes	No	Yes
Spinal canal stenosis	Yes	Yes	Yes
Biceps tendon rupture	No	No	No

DPD, 3,3-diphosphono-1,2-propanodicarboxylic acid; LV, left ventricular; MI, myocardial infarction; NSVT, non-sustained ventricular tachycardia; NTproBNP, N-terminal pro-B-type natriuretic peptide.; T2DM, type 2 diabetes mellitus.

## Discussion

Previously considered a rare disease, ATTR-CM has been shown to be prevalent in a range of cardiac disease cohorts.^19^ Diagnosis is aided by certain ‘red flag’ features including a history of carpal tunnel syndrome or spinal canal stenosis, low limb lead voltages on ECG and echocardiographic features such as concentric LV hypertrophy and reduced global longitudinal ventricular strain with apical sparing.^20 21^

Conduction disease is a well-recognised feature of ATTR-CM, with just under 1 in 10 newly diagnosed patients requiring PPM implantation.^9^ Conversely, the prevalence of ATTR-CM in patients requiring a PPM is less certain, with only a single study to date showing a prevalence of less than 2% in patients over 60 years of age requiring pacing.^22^ While this study’s inclusion criteria allowed recruitment of younger patients, the mean age was higher than that seen in our study. There was, however, a lower proportion of males (56% vs 78%) which may explain the higher prevalence of ATTR-CM in our cohort. Of note, patients with a positive DPD scan all had LV hypertrophy and a prior history of carpal tunnel syndrome or spinal canal stenosis, illustrating the importance of such diagnostic red flags in directing the investigation of high-grade AV block. In comparison, the prevalence of diagnostic red flags was very low in patients returning a negative DPD scan.

Tafamidis, a TTR-stabilising agent, has received a class 1B recommendation in the European Society of Cardiology heart failure guidelines for use in ATTR-CM patients with NYHA class I or II symptoms.^23^ Clinical trials examining other TTR-stabilising agents, gene silencers and gene editing technologies are showing promise.^24 25^ This prospective study highlights the importance of maintaining a high-degree of suspicion for ATTR-CM in older patients requiring a PPM so that they too can be offered disease-modifying therapy.

To maximise the potential of new therapies, early diagnosis of ATTR-CM is key. This is particularly the case in the presence of high-degree AV block which appears to herald a poorer prognosis in patients with ATTR-CM. A recent study found that the presence of a PPM at baseline assessment was associated with a significantly higher risk of heart failure admissions and a trend towards a higher rate of mortality.^26^ In a second study, death from any cause occurred in 83% of ATTR-CM patients who developed high-degree AV block during follow-up vs 29% without conduction disease. High-degree AV block was significantly associated with all-cause mortality on univariable analysis but did not reach statistical significance in a multivariable model.^9^

## Limitations

The study is limited by the small cohort size. The low recruitment rate can be attributed to the frailty of the target population in combination with the occurrence of the COVID-19 pandemic during the study period.

## Conclusion

Eight per cent of individuals between 70 and 85 years of age undergoing PPM implantation for high-degree AV block have ATTR-CM.

## Data Availability

All data relevant to the study are included in the article or uploaded as online supplemental information.
